# Sino-Cranial Infection Caused by *Curvularia spicifera* in a Young Adult Without Overt Immunosuppression: A Case Report and Review of the Literature

**DOI:** 10.3390/microorganisms14102245

**Published:** 2026-10-03

**Authors:** Arianna Emiliozzi, Alessandro Capone, Marina Selleri, Carlo Giacomo Palazzo, Antonella Vulcano, Chiara Temperoni, Alessandra Lodi, Davide Luglietto, Assunta Gallo, Riccardo Antonio Ricciuti, Carla Fontana, Stefania Cicalini

**Affiliations:** 1 Systemic and Immune Depression-Associated Infections Unit, National Institute for Infectious Diseases “Lazzaro Spallanzani”, IRCCS, 00149 Rome, Italy; arianna.emiliozzi@inmi.it (A.E.); stefania.cicalini@inmi.it (S.C.); 2 Laboratory of Microbiology and Biobank, National Institute for Infectious Diseases “Lazzaro Spallanzani”, IRCCS, 00149 Rome, Italycarlo.palazzo@inmi.it (C.G.P.); antonella.vulcano@inmi.it (A.V.);; 3 Department of Neurosurgery, Azienda Ospedaliera San Camillo Forlanini, 00152 Rome, Italy; dluglietto@scf.rm.it (D.L.);; 4 Laboratory of Microbiology, Azienda Ospedaliera San Camillo Forlanini, 00152 Rome, Italy

**Keywords:** *Curvularia spicifera*, epidural empyema, allergic fungal rhinosinusitis, phaeohyphomycosis, marijuana, cannabis, dog training, dematiaceous fungi, voriconazole, amphotericin B

## Abstract

*Curvularia* species are melanized dematiaceous molds widely distributed in soil, plants, and decaying organic matter. Human infections are uncommon and typically involve the paranasal sinuses, skin, cornea, or respiratory tract, whereas central nervous system (CNS) involvement is rare. We report a locally invasive sino-cranial infection caused by *Curvularia spicifera* in a 20-year-old immunocompetent man with chronic sinusitis and features consistent with allergic fungal rhinosinusitis (AFRS). Brain magnetic resonance imaging revealed acute-on-chronic sinusitis complicated by a frontal sinus mucocele with orbital and intracranial extension. Management involved urgent neurosurgical source control followed by endoscopic sinonasal surgery. *C. spicifera* was repeatedly isolated from independent deep surgical specimens. The isolate was identified as *Curvularia spicifera* using a polyphasic approach integrating morphological, proteomic (MALDI-TOF MS), and molecular evidence. Histopathological examination demonstrated mixed neutrophilic and eosinophilic inflammation consistent with AFRS. Antifungal treatment was initiated with liposomal amphotericin B plus voriconazole; amphotericin B was subsequently discontinued because of severe hypokalemia, whereas voriconazole was continued under therapeutic drug monitoring. At the time of writing, the patient had completed three months of voriconazole therapy, with progressive clinical improvement and good tolerability, and was awaiting further radiological follow-up to guide the decision on treatment duration. Review of published cases indicates that contiguous spread from chronic sinonasal disease represents a frequent pathogenic mechanism of CNS *Curvularia* infection and that severe disease may occur even in the absence of overt immunosuppression. This case highlights the importance of early multidisciplinary management, aggressive surgical source control, accurate microbiological identification, and prolonged mold-active antifungal therapy with careful monitoring for treatment-related toxicity.

## 1. Introduction

*Curvularia* is a filamentous dematiaceous fungus found in soil and plant material, especially in tropical and subtropical environments [1]. Human infections caused by *Curvularia* species are uncommon and are generally classified within the spectrum of phaeohyphomycosis [2], a group of infections caused by melanized fungi. Reported clinical manifestations include allergic and invasive fungal rhinosinusitis [3], keratitis, cutaneous and subcutaneous infections, respiratory disease, disseminated infection [4], and rare central nervous system (CNS) involvement.

Unlike many invasive mold infections that predominantly affect severely immunocompromised hosts, *Curvularia* infections have frequently been reported in immunocompetent individuals [5]. In CNS involvement, morbidity and mortality remain high. Most reported cases appear to arise either from contiguous extension of chronic or invasive sinonasal disease or, less commonly, from hematogenous spread after inhalational or cutaneous acquisition. *Curvularia spicifera*, formerly classified within *Bipolaris spicifera*, has been implicated in invasive and non-invasive human disease [3,6], including sino-orbital and CNS presentation [7].

The taxonomy of *Curvularia* has undergone substantial revision over the last decade [8]. Multilocus phylogenetic analyses have shown that several species that are clinically significant and were previously classified in the genus *Bipolaris*, such as *B. spicifera* and *Bipolaris hawaiiensis*, are part of the *Curvularia* complex [9]. This taxonomic reorganization has significant clinical implications, as historical reports of CNS and sinus infections attributed to *B. spicifera* should now be interpreted as *C. spicifera* infections. Additionally, morphological identification alone may be unreliable due to morphological overlap among dematiaceous fungi. Thus, molecular characterization is increasingly important for species-level identification [3,10].

Predisposing factors described in the literature include chronic sinusitis, allergic fungal rhinosinusitis (AFRS) [11], previous sinonasal surgery, immunosuppression, corticosteroid exposure, diabetes, exposure to contaminated organic material, and inhaled marijuana [12]. These conditions represent distinct clinicopathological entities. Fungal colonization refers to recovery of a fungus without evidence of associated tissue disease, whereas a fungal ball is characterized by a non-invasive accumulation of fungal elements within a sinus cavity. AFRS is an eosinophil-dominant inflammatory disorder associated with allergic mucin and fungal material, without histological evidence of tissue invasion. Invasive fungal rhinosinusitis, by contrast, involves extension into the sinonasal mucosa, bone, orbit, vascular structures, or intracranial compartment. Distinguishing among these entities requires integration of microbiological, histopathological, radiological, and operative findings.

However, the factors that allow progression from localized sinonasal disease to invasive sino-cranial infection in apparently immunocompetent hosts remain poorly understood.

Due to the rarity of CNS involvement, optimal management remains poorly standardized and generally relies on aggressive surgical debridement combined with prolonged systemic antifungal therapy [13]. Here, we report a case of an intracranial infection caused by *C. spicifera* in a young immunocompetent man and review the available literature on CNS and intracranial *Curvularia* infections, including cases historically reported under the Bipolaris nomenclature, identified through a narrative review of the literature in PubMed and Embase. Host-related, local sinonasal, and environmental factors that may have contributed to the development of invasive sino-cranial infection in an apparently immunocompetent patient were also discussed.

## 2. Case Report

A 20-year-old immunocompetent man from Central Italy whose medical history was notable only for chronic sinusitis and who was not taking any regular medications was referred to the emergency department of a tertiary care hospital after an outpatient non-contrast brain computed tomography (CT) scan revealed acute-on-chronic frontal and ethmoidal sinusitis complicated by a 26 mm frontal sinus mucocele. The lesion was associated with erosion of the posterior sinus wall and contiguous extension into the right frontal lobe and superior orbit (Figure 1A,B).

The patient reported a 6-month history of anosmia and ageusia, followed by progressive headache, right orbital pain, and periorbital swelling. He denied intranasal drug use but reported occasional inhaled marijuana use. Occupational history was notable for outdoor work as a dog trainer, with frequent exposure to soil, grass, dust, and rural environments. He also reported consuming approximately one alcoholic drink per day.

No underlying immunosuppressive conditions or other recognized risk factors for invasive fungal infection were identified.

On admission, the patient was in good general condition, afebrile, alert, cooperative, and fully oriented, with no focal neurological deficits. Laboratory tests were unremarkable except for leukocytosis (15,000 cells/mm^3^) with neutrophilia, while C-reactive protein was within the normal range.

Contrast-enhanced magnetic resonance imaging (MRI) confirmed acute-on-chronic frontal and ethmoidal sinusitis complicated by a 26 mm frontal sinus mucocele with erosion of the posterior sinus wall and contiguous extension into the right frontal lobe and superior orbit. The intracranial lesion demonstrated imaging characteristics consistent with an epidural empyema associated with surrounding vasogenic edema (Figure 2). No additional intracranial lesions were identified.

Because of the extent of intracranial involvement and the risk of neurological deterioration, urgent neurosurgical intervention was undertaken. Intraoperative findings demonstrated extensive frontal sinus destruction with erosion of the posterior sinus wall, skull base osteolysis involving the ethmoid and orbital roofs, and inflammatory tissue densely adherent to the dura overlying the frontal lobe. A bifrontal craniotomy with frontal sinus cranialization and extensive debridement was performed. Additional purulent material and dense mucoid contents consistent with a frontal sinus mucocele were identified and removed. Multiple deep specimens obtained during surgery were submitted for microbiological and histopathological analyses. The extensive inflammatory and osteolytic process confirmed the locally invasive nature of the disease and allowed adequate surgical source control.

One week later, after stabilization of the neurological condition, endoscopic sinonasal surgery was performed to achieve complete debridement and further source control, including ethmoidectomy and excision of polypoid inflammatory tissue involving the frontal recess and ethmoidal cells. Additional tissue specimens were submitted independently for microbiological and histopathological analyses.

Laboratory investigations excluded major causes of immunodeficiency. HIV serology and chronic viral hepatitis screening were negative. As part of the immunological work-up performed to investigate possible underlying predisposing conditions, serum immunoglobulins were assessed. Total serum IgE was elevated (1002 kU/L), while a mild reduction in IgG was detected (652 mg/dL; reference range 700–1600 mg/dL). This isolated mild IgG reduction was not considered indicative of a clinically significant immunodeficiency. Chest radiography and abdominal ultrasonography did not reveal additional infectious foci. Serum β-D-glucan and galactomannan tests were negative.

Pending microbiological results, empirical broad-spectrum antibacterial therapy with meropenem at CNS dosage and linezolid was initiated by the neurosurgical team. Linezolid, however, was discontinued early because of severe gastrointestinal intolerance.

Microbiological cultures from the first intraoperative specimen yielded *Curvularia* species and *Bacillus* spp. Cultures from the second surgical specimen again isolated *Curvularia* species, subsequently identified as *C. spicifera*, together with coagulase-negative staphylococci. The repeated isolation of *Curvularia* from deep surgical specimens supported its etiologic role.

Species identification was initially obtained by matrix-assisted laser desorption ionization time-of-flight mass spectrometry (MALDI-TOF MS) using the MALDI Biotyper Sirius system (Bruker Daltonics GmbH & Co. KG, Bremen, Germany), which identified the isolate as *Curvularia spicifera* with a secure species-level score of 2.0. Given the rarity of the isolate and the invasive nature of the infection, identification was subsequently further evaluated by PCR amplification and Sanger sequencing of the D1/D2 domain of the 28S ribosomal RNA gene using primers P1 (5′-ATCAATAAGCGGAGGAAAAG-3′) and P2 (5′-CTCTGGCTTCACCCTATTC-3′). PCR reactions were carried out in a final volume of 25 μL containing 1× PCR buffer, dNTPs (1.25 mM each), 10 pmol of each primer, Taq DNA polymerase (Takara Bio Inc., Shiga, Japan), and nuclease-free water. Amplification consisted of 40 cycles of denaturation at 94 °C for 45 s, annealing at 50 °C for 45 s, and extension at 72 °C for 1 min. PCR products were purified using ExoSAP-IT™ PCR Product Cleanup Reagent (Applied Biosystems, Foster City, CA, USA) and subsequently sequenced in both directions on an Applied Biosystems 3500xL Genetic Analyzer (Thermo Fisher Scientific, Waltham, MA, USA). Forward and reverse sequences were assembled into a consensus sequence using BioEdit version 7.7.1.0. The obtained partial D1/D2 LSU sequence (359 nucleotides) was analyzed using both the NCBI BLAST tool version BLASTN 2.17.0 and the MycoBank pairwise alignment platform against multiple curated fungal databases, including FunCBS, UNITE, SILVA_LSU, RefSeq, ITS/EF1 MycologyLab, PasteurYeasts/MOLDS, fusarioidID, and MIRRI. The sequence showed 100% identity over 359 nucleotides and 99% query coverage with *Curvularia spicifera* reference sequences. Acknowledging the recognized taxonomic complexity of the *Curvularia/Bipolaris* complex and the limitations of relying on a single relatively short genetic marker, species-level assignment was not based on sequence data alone. Instead, a polyphasic approach was adopted, integrating phenotypic, proteomic, and molecular evidence. The isolate displayed macroscopic and microscopic features consistent with *Curvularia* spp. MALDI-TOF MS independently identified the isolate as *C. spicifera*, and molecular analysis supported the same identification. Therefore, the identification of the isolate as *Curvularia spicifera* was based on the convergence of independent phenotypic, proteomic, and molecular findings rather than on D1/D2 sequencing alone.

Antifungal susceptibility testing was performed using the Sensititre™ YeastOne™ colorimetric broth microdilution system (Thermo Fisher Scientific, Waltham, MA, USA) according to the manufacturer’s instructions for mold susceptibility testing. Plates were incubated at 35 °C and examined after 24, 48, and 72 h. MIC values were determined by visual assessment of the colorimetric endpoint and were defined as the lowest antifungal concentration preventing the color change in the growth indicator associated with fungal growth. Because no CLSI or EUCAST clinical breakpoints or species-specific epidemiological cutoff values are currently available for *Curvularia spicifera*, MIC values were reported without categorical interpretation. Previous studies have shown good agreement between the Sensititre YeastOne system and CLSI broth microdilution methodologies for antifungal susceptibility testing of filamentous fungi [14,15].

The isolate exhibited low MIC values for amphotericin B, itraconazole, and posaconazole, whereas higher MIC values were observed for fluconazole and echinocandins.

On Sabouraud dextrose agar, the isolate produced rapidly growing colonies that were initially grey-white and cottony. Over time, the colonies became dark olive-brown to black and developed a darkly pigmented reverse (Figure 3 and Figure 4). Microscopic examination of a lactophenol cotton blue preparation revealed septate dematiaceous hyphae and brown multicellular conidia with morphological features consistent with *Curvularia* species. These combined macroscopic and microscopic findings supported the identification of a dematiaceous mold belonging to the genus *Curvularia.* However, given the recognized morphological overlap within the *Curvularia-Bipolaris* complex, species-level identification was supported by the combined results of colony morphology, MALDI-TOF MS analysis, and D1/D2 sequencing.

Table 1 summarizes the MIC values obtained by antifungal susceptibility testing of the *Curvularia* isolates recovered from the first and second clinical samples.

Antifungal susceptibility testing was performed using the Sensititre™ YeastOne™ colorimetric broth microdilution system according to the manufacturer’s instructions for mold susceptibility testing. As no CLSI or EUCAST clinical breakpoints or species-specific epidemiological cutoff values (ECVs) are currently available for *C. spicifera*, MICs are reported without categorical interpretation. The discrepancy between the initial and reference laboratory voriconazole MICs highlights the challenges of antifungal susceptibility testing for uncommon dematiaceous fungi. Notably, susceptibility testing was repeated twice at the reference laboratory and yielded the same voriconazole MIC (2 µg/mL) in both determinations. While itraconazole, posaconazole, and amphotericin B showed closely comparable MIC values between the two laboratories, some variability was also observed for fluconazole and the echinocandins. Similar variability in azole susceptibility has been reported among clinically relevant *Curvularia* isolates, including members of the *C. spicifera* clade. Consequently, antifungal selection was based on the integration of available in vitro susceptibility data, pharmacokinetic/pharmacodynamic (PK/PD) principles, therapeutic drug monitoring (TDM), expected CNS drug exposure, and the patient’s clinical and radiological response [3,14,16,17].

Histological examination of neurosurgical and otorhinolaryngological specimens documented mixed neutrophilic and eosinophilic inflammation. In particular, nasal tissue showed polypoid sinonasal mucosa with a dense mixed inflammatory infiltrate rich in eosinophilic granulocytes, findings compatible with AFRS, although formal diagnostic criteria could not be fully documented.

Four days after the first surgical intervention, following the microbiological finding, an Infectious Diseases consultation was requested, and combined antifungal therapy with intravenous liposomal amphotericin B and oral voriconazole was initiated, while susceptibility testing was still pending. Meropenem was discontinued after 10 days because of progressive liver transaminase elevation and because repeated *Curvularia* isolation supported fungal infection as the primary process. After two weeks of combined antifungal therapy, the patient developed severe hypokalemia (1.8 mmol/L) without electrocardiographic abnormalities or clinical manifestations. Liposomal amphotericin B was discontinued, and aggressive electrolyte replacement resulted in progressive normalization. Voriconazole TDM was performed, and dose escalation to 600 mg/day was required to achieve therapeutic concentrations. The patient remained afebrile and clinically stable, with progressive improvement, and was discharged 22 days after neurosurgical intervention on oral voriconazole.

Follow-up brain and maxillofacial MRI performed one month after neurosurgery showed heterogeneous frontal sinus material and meningeal enhancement, interpreted as post-inflammatory changes in the context of a favorable clinical evolution. Despite the favorable radiological findings, given the type of infection and the extent of CNS involvement, we decided to continue voriconazole therapy. At the time of writing, the patient had completed more than three months of treatment, with progressive clinical improvement and good clinical and laboratory tolerability of voriconazole. A further follow-up brain MRI is scheduled at four months after surgery and will guide the decision on treatment discontinuation, together with the overall clinical course, drug exposure, and tolerability, in the absence of established guidelines defining the optimal duration of antifungal therapy for CNS *Curvularia* infections.

## 3. Discussion

The present case describes a locally invasive CNS fungal infection caused by *C. spicifera* in an immunocompetent host. Its main features of interest include the probable contiguous spread from chronic sinonasal disease, the coexistence of histopathological findings suggestive of AFRS phenotype, and the repeated isolation of *C. spicifera* from deep surgical specimens supported by molecular evidence. Together, these findings support a true invasive infection rather than environmental contamination and further demonstrate that *Curvularia* species may cause severe CNS disease even in the absence of overt immunosuppression.

CNS and intracranial infections caused by *Curvularia* species represent a rare manifestation within the broader spectrum of dematiaceous fungal infections of the central nervous system, which are more commonly associated with neurotropic pathogens such as *Cladophialophora bantiana* [2].

Since the first intracerebral infection attributed to a species currently classified within the genus *Curvularia* in 1973 [18], an increasing number of cases have been described, likely reflecting both improved recognition of *Curvularia* as a human pathogen and advances in microbiological identification techniques.

As shown in Table 2, the clinical spectrum of CNS Curvularia infection is remarkably broad, including brain abscess, cerebritis, meningitis, cranial-base mass-like disease, and intracranial extension of invasive sinusitis.

In parallel, the taxonomy of the genus has undergone substantial revision. Multilocus phylogenetic studies have shown that several clinically relevant fungi, including *B. spicifera* and *B. hawaiiensis*, are part of the *Curvularia* complex. Consequently, a significant proportion of historical reports of CNS and sinus infections attributed to *Bipolaris* species would currently be classified as *Curvularia* infections. This taxonomic reclassification is important to consider when reviewing older clinical series and interpreting published outcome data [9].

Both immunocompromised and immunocompetent hosts have been affected by CNS *Curvularia* infections. Reported risk groups include patients with solid or hematological malignancies, transplant recipients, individuals receiving immunosuppressive therapy, and premature neonates. However, numerous cases of intracerebral and rhinosinus-related infection have also occurred in otherwise healthy individuals, supporting a relevant role for local predisposing factors and environmental exposure in disease pathogenesis.

The literature identifies three main clinical patterns of CNS *Curvularia* infection: (i) contiguous extension from chronic or invasive sinonasal disease, often involving the frontal sinus, skull base, or orbit [37]; (ii) infection occurring in the setting of significant immunosuppression, such as solid-organ transplantation, hematological malignancies, or prolonged corticosteroid therapy [31]; and (iii) disease potentially related to inhalational exposure to contaminated organic material, including marijuana [33], particularly in the presence of local or systemic predisposing factors. The present case shares features with both the first and third patterns, given the longstanding sinonasal disease and the presence of potential environmental exposures.

Notably, the clinical course was slowly progressive, with months of anosmia, ageusia, headache, orbital pain, and periorbital swelling before diagnosis, mirroring several previously reported cases in which sinonasal manifestations preceded CNS involvement by *weeks* or months.

These observations support a pathogenic model in which inhaled *Curvularia* conidia colonize chronically inflamed sinonasal mucosa, particularly in the presence of an allergic inflammatory environment [38]. Elevated serum IgE levels and eosinophil-rich polypoid mucosa are consistent with an AFRS-like phenotype. Recent evidence has further strengthened the association between *C. spicifera* and allergic fungal rhinosinusitis in immunocompetent individuals. Ferraro et al. described two cases of non-invasive fungal rhinosinusitis in which diagnosis relied on the integration of radiological findings, eosinophil-rich inflammatory mucosa, fungal culture, and molecular identification. Their observations support the concept that *C. spicifera* may persist within a chronic allergic inflammatory environment and contribute to progressive sinonasal disease even in the absence of an overt immunosuppression phenotype [3]. Chronic obstruction together with persistent fungal-allergic inflammation may have promoted mucocele formation, progressive local expansion, bone erosion [39], orbital involvement, and ultimately contiguous spread to the frontal lobe [40]. Although the patient was not overtly immunocompromised, subtle host-related factors, including mild IgG reduction and pronounced type 2/eosinophilic inflammation, may have increased susceptibility to locally invasive disease.

Environmental exposure deserves consideration in the present case. *Curvularia* species are ubiquitous environmental fungi that are widely distributed in soil, grass, decaying vegetation, and plant debris. Although this widespread distribution may complicate distinguishing infection from contamination, the repeated isolation of *C. spicifera* from multiple deep surgical specimens obtained during separate procedures, together with consistent radiological findings and histopathological evidence of fungus-associated inflammation, strongly supports its role as the causative pathogen rather than an incidental colonizer.

Based on the patient’s occupational and recreational history, we hypothesized that two kinds of exposures may have increased his predisposition to developing the infection: inhalational marijuana use and repeated outdoor exposure.

Recreational marijuana smoking represents a plausible source of inhalational fungal exposure, given the well-documented contamination of cannabis products by viable fungal spores, including *Aspergillus*, *Curvularia*, and other environmental molds [41].

Marijuana use has been associated with invasive fungal infections, particularly aspergillosis in susceptible hosts, presumably through inhalation of contaminated plant material and subsequent respiratory or sinonasal colonization. Notably, CNS *Curvularia* infections have been reported in individuals with heavy marijuana use, including a patient receiving chronic corticosteroid therapy for neurosarcoidosis who developed a brainstem abscess [12,28]. Although these observations do not establish a causal relationship, they highlight the importance of carefully assessing environmental and recreational exposures in patients presenting with uncommon mold infections.

In addition, the patient’s occupation as an outdoor dog trainer may have increased exposure to environmental reservoirs of *Curvularia* through repeated contact with soil, grass, dust, and organic material. While *Curvularia* infections have been described in dogs [42,43], most commonly as post-traumatic cutaneous infections and keratitis [44], no epidemiologic evidence currently links dog-training activities to human *Curvularia* sinusitis or CNS infection. Nevertheless, repeated exposure to outdoor environments may have increased opportunities for inhalational acquisition and should be regarded as a potential contributing factor rather than an established risk factor.

Figure 5 illustrates the hypothetical pathogenetic model for the sino-cranial *Curvularia* infection observed in the present case. The model hypothesizes inhalational acquisition from contaminated organic material and environmental exposure to soil and grass, followed by sino-nasal colonization in the setting of chronic AFRS. Subsequent local progression may have led to mucocele formation, erosion, intraorbital spread, and ultimately contiguous extension to the frontal lobe with empyema development.

Accurate microbiological identification is essential for the management of infections caused by dematiaceous fungi. Morphological differentiation among members of the *Curvularia–Bipolaris–Cochliobolus* complex can be challenging because of the frequent overlap in colony morphology and microscopic features. In the present case, species identification obtained by MALDI-TOF MS was further supported by sequencing of the D1/D2 domain of the 28S rRNA gene, providing complementary taxonomic information in support of the MALDI-TOF MS result. The combination of proteomic and molecular approaches is particularly valuable for uncommon molds such as *Curvularia*, for which historical taxonomic revisions and phenotypic overlap may complicate conventional identification. Molecular sequencing has therefore become increasingly important for accurate species-level identification and for clarifying epidemiological associations. In the present case, species assignment was based on the integration of morphological, proteomic, and molecular findings rather than on D1/D2 sequencing alone. In recent years, these approaches have substantially improved recognition of *C. spicifera* as an emerging opportunistic human pathogen [3].

An additional finding of interest was the marked difference between the voriconazole MIC obtained in the local laboratory and that reported by the reference laboratory (0.12 vs. 2 μg/mL). Although the reasons for this discrepancy cannot be determined retrospectively, susceptibility testing of uncommon dematiaceous fungi remains challenging, and inter-laboratory variation has been described. Moreover, antifungal susceptibility profiles within the genus *Curvularia* are heterogeneous. In a large study of molecularly identified clinical isolates, voriconazole and itraconazole showed limited overall activity against several *Curvularia* species [45], while earlier investigations reported elevated azole fungicidal endpoints in some isolates belonging to the *B. spicifera*/*C. spicifera* group [46]. As no CLSI or EUCAST clinical breakpoints are currently available for *C. spicifera*, MIC values should be interpreted cautiously and integrated with clinical evolution, drug exposure, and site of infection. In our patient, the decision to continue voriconazole therapy was supported by its favorable CNS penetration, TDM results, and the progressive clinical and radiological improvement observed during follow-up.

CNS infection caused by *Curvularia* is associated with substantial morbidity and mortality, even among immunocompetent individuals. Reported cases include both therapeutic failures and late relapses, highlighting the aggressive nature of the disease and the risk of unfavorable outcomes.

Management of CNS *Curvularia* infections remains particularly challenging because of the limited number of effective antifungal drugs, the absence of organism-specific clinical breakpoints, and the lack of randomized studies or evidence-based recommendations regarding the duration of therapy.

Consequently, current practice relies largely on published case experience and expert guidance on phaeohyphomycosis, which emphasize the importance of aggressive source control [32], repeated debridement when required, and prolonged systemic antifungal therapy; amphotericin B, itraconazole, voriconazole, and combination regimens have all been used in published *Curvularia* infections (Table 2).

Given the severity of CNS *Curvularia* infections and the risk of treatment failure or late relapse, selection of an effective and tolerable antifungal regimen is critical. Historically, amphotericin B, either as monotherapy or in combination with a triazole, has represented the cornerstone of treatment [1,14], while itraconazole was widely used in earlier years when alternative azoles were not available. Voriconazole has subsequently emerged as an attractive option because of its oral formulation and favorable CNS penetration; however, its use requires TDM and careful surveillance for hepatotoxicity, neurotoxicity, visual disturbances, photosensitivity, and drug–drug interactions.

In our patient, amphotericin B had to be discontinued because of severe hypokalemia, underscoring the practical challenges and toxicity-related limitations of prolonged combination therapy.

Isavuconazole may represent a promising alternative for long-term treatment owing to its favorable pharmacokinetic profile, good tolerability, and demonstrated in vitro activity against dematiaceous fungi [47]. Clinical experience with *Curvularia* infections remains limited, although sporadic cases have been reported. Notably, Cornely et al. [48] described successful treatment of disseminated pulmonary and cutaneous *Curvularia lunata* infection in a patient with hematological malignancy, with a favorable response after 52 days of isavuconazole therapy.

Despite advances in antifungal therapy, timely and aggressive surgical source control, including complete excision or debridement whenever anatomically feasible, remains a cornerstone of treatment and should be combined with prolonged systemic antifungal therapy to optimize clinical outcomes. In our patient, urgent neurosurgical intervention was considered necessary because of the site and extent of intracranial involvement and the risk of neurological deterioration; however, the timing and extent of surgery should be individualized according to the clinical and radiological features of each case.

Regarding the optimal duration of antifungal treatment for CNS *Curvularia* infections, no standardized recommendations are currently available. Treatment courses reported in the literature vary considerably, reflecting the heterogeneity of clinical presentation, the extent of intracranial involvement, the immune status of the host, and the completeness of surgical debridement. Among the published case reports reviewed, treatment duration ranged from a few weeks to 12 months; however, the exact timing of treatment discontinuation and long-term follow-up were inconsistently reported. Consequently, the available evidence remains insufficient to define the optimal duration of antifungal treatment or to support evidence-based recommendations [49].

Nevertheless, prolonged therapy is generally considered necessary because of the severity of CNS infection and the potential risk of persistence or relapse. Treatment duration should therefore be individualized and guided by clinical evolution, serial radiological findings, the adequacy of surgical source control, and drug tolerability [49].

Given the extent of CNS involvement, in our patient, voriconazole treatment was continued beyond three months. The favorable safety profile observed in this patient allowed prolonged therapy while serial imaging was used to monitor disease evolution and guide future treatment discontinuation.

Overall, awareness of potential risk factors for *Curvularia* infection and their careful investigation during the diagnostic workup may facilitate earlier recognition of the disease and prompt initiation of appropriate management.

This case reinforces the importance of multidisciplinary care involving infectious disease specialists, neurosurgeons, otolaryngologists, microbiologists, radiologists, and pathologists. Repeated isolation from deep specimens, histopathologic correlation, species-level identification, antifungal susceptibility testing, and imaging follow-up are all essential for establishing the diagnosis and guiding therapy.

## 4. Conclusions

This case highlights that *C. spicifera* can cause severe, locally invasive sino-cranial infection even in young individuals without overt immunosuppression. Chronic sinusitis/AFRS, inhalational marijuana use, and repeated occupational outdoor exposure were identified as potential local or exposure-related factors in this patient; however, their possible role in the development of invasive infection should be considered hypothetical and cannot be inferred from a single case. The available evidence underscores the importance of maintaining a high index of suspicion, obtaining a detailed environmental and recreational exposure history, and achieving prompt surgical source control combined with microbiological identification supported by phenotypic and molecular methods. Given the potential for treatment failure, toxicity, and late relapses, prolonged antifungal therapy and careful long-term clinical and radiological follow-up remain essential components of management.

## Figures and Tables

**Figure 1:**
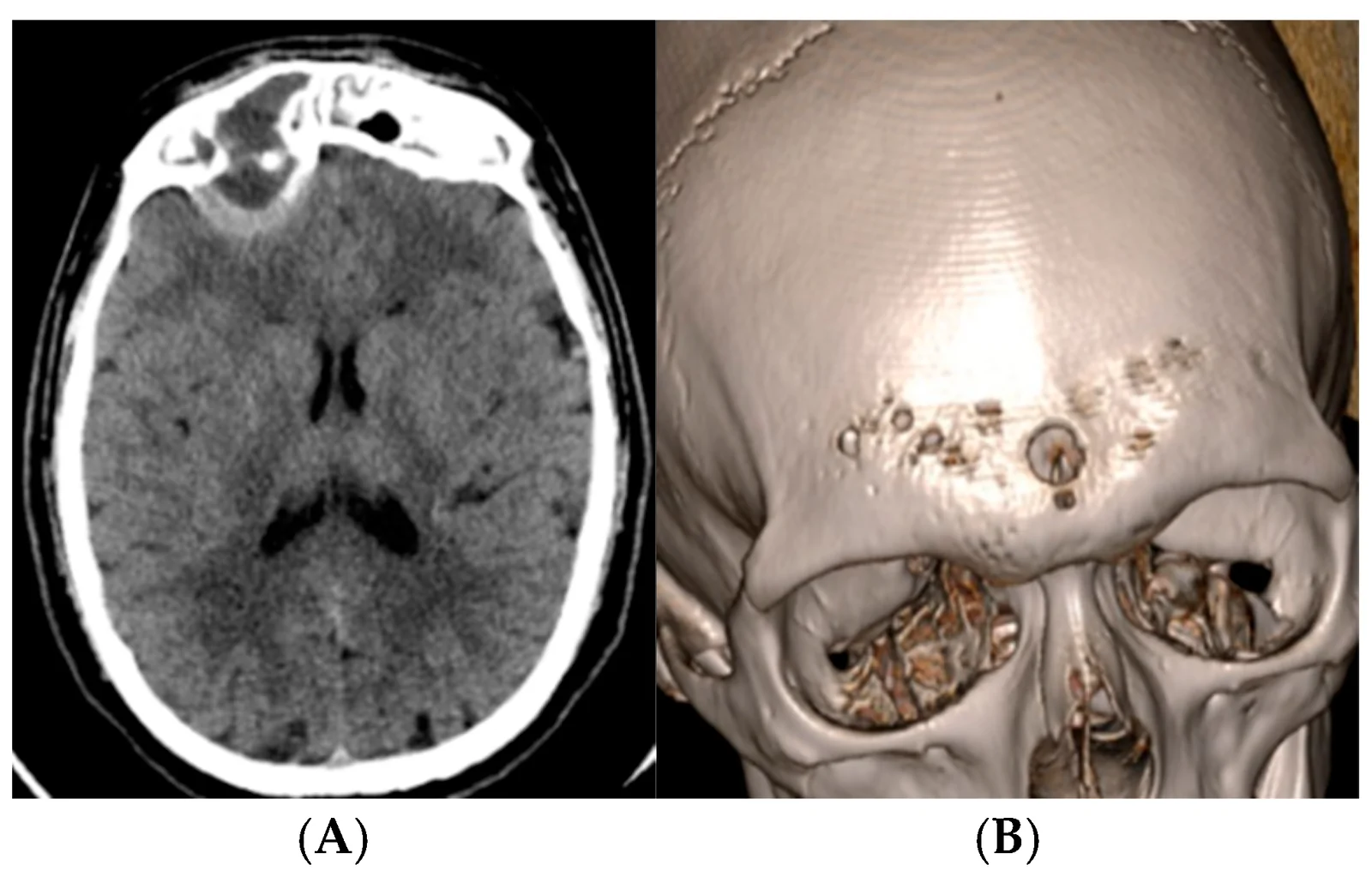
(**A**) Axial brain CT scan showing a frontal sinus mucocele with erosion of the posterior sinus wall and direct extension into the right frontal lobe. (**B**) Three-dimensional CT reconstruction confirming erosion of the outer table of the frontal bone.

**Figure 2:**
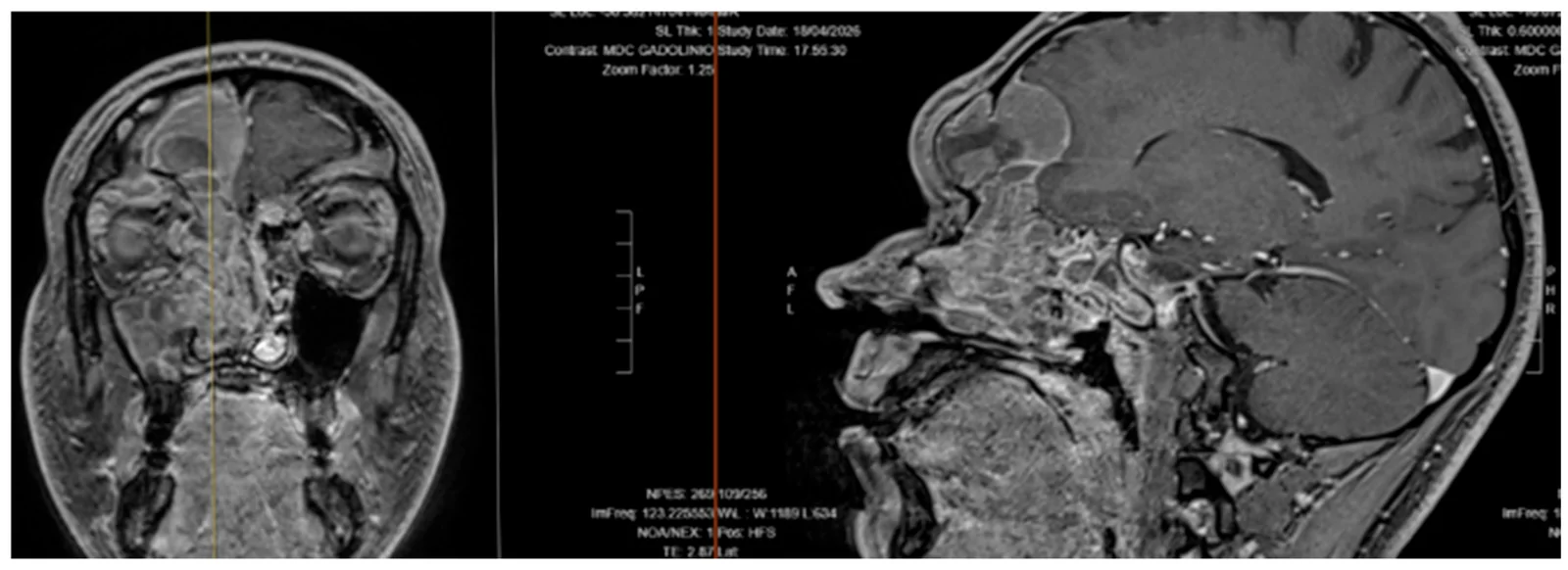
Coronal and sagittal contrast-enhanced T1-weighted MRI demonstrating ethmoidal, maxillary, and frontal sinusitis associated with a right frontal epidural empyema.

**Figure 3:**
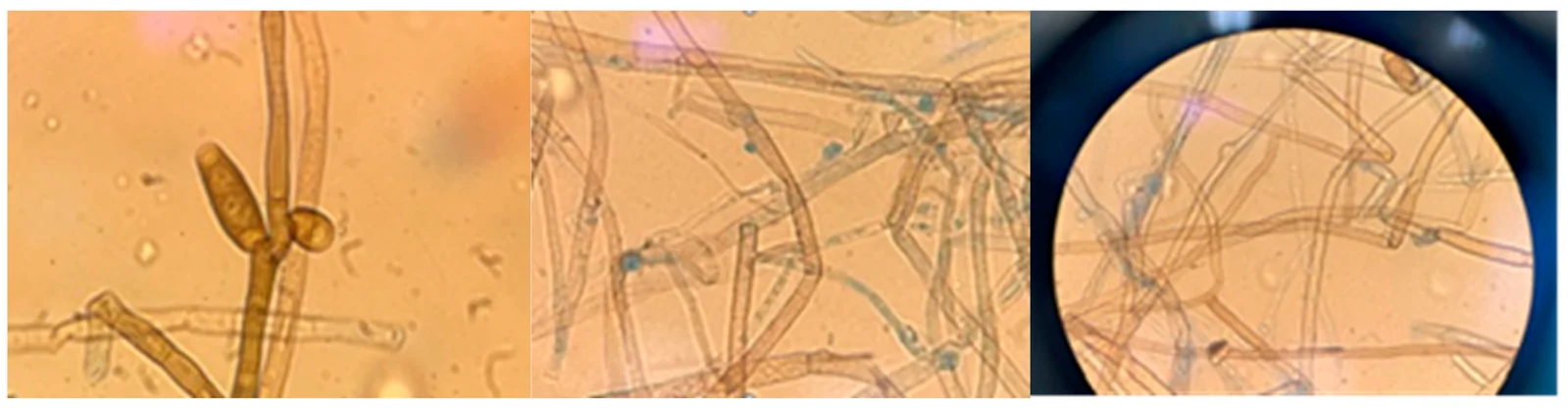
Microscopic morphology of *Curvularia spicifera* after lactophenol cotton blue staining. The preparation shows septate dematiaceous hyphae and brown multicellular conidia that are cylindrical to slightly curved, consistent with the morphology of *Curvularia* species.

**Figure 4:**
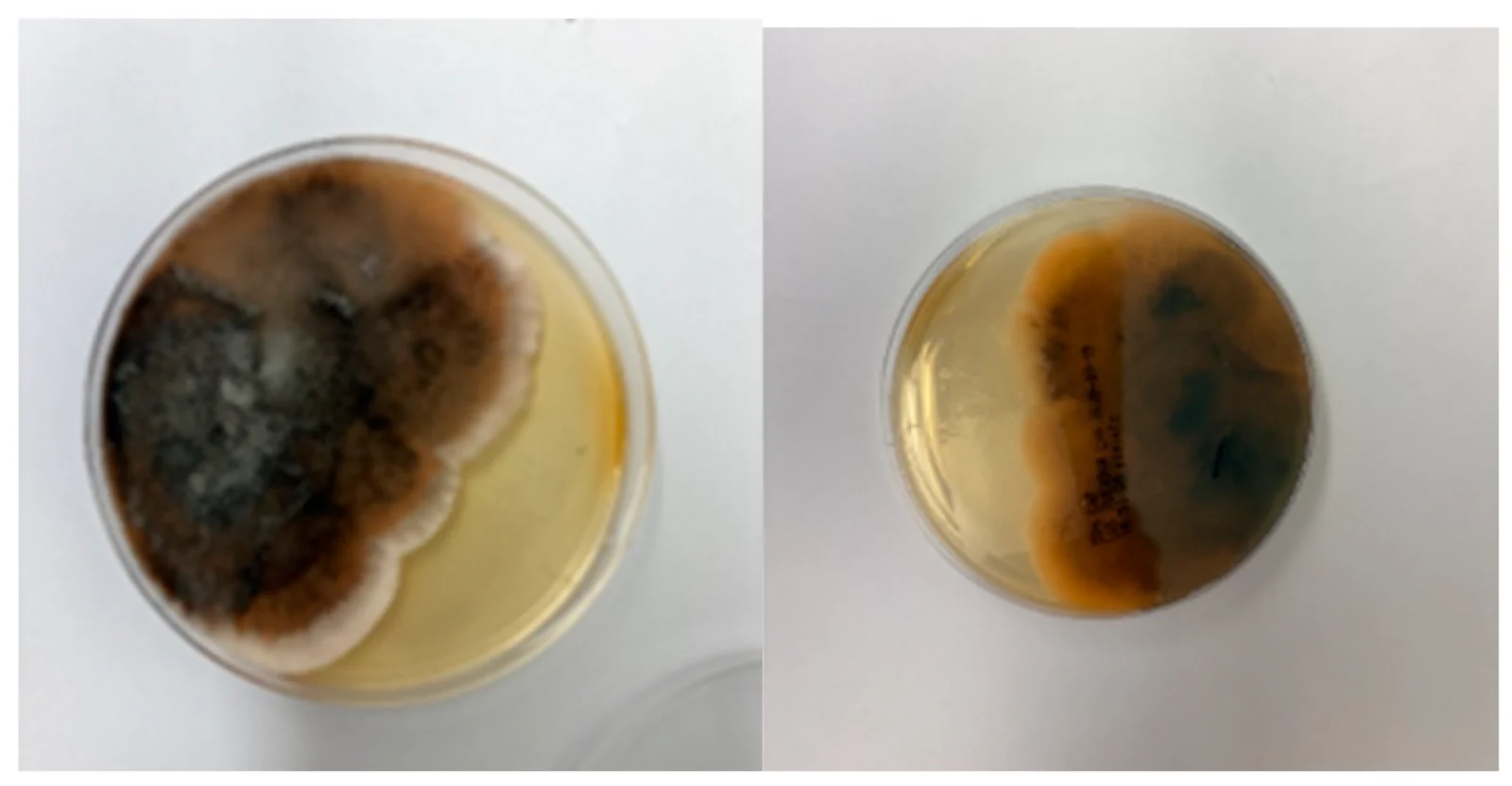
Macroscopic appearance of *Curvularia spicifera*. Colonies grown on Sabouraud dextrose agar showed rapid growth with a cottony to floccose pale grey/olivaceous-grey surface that progressively became olivaceous-brown to blackish brown, with a dark brown to black reverse.

**Figure 5:**
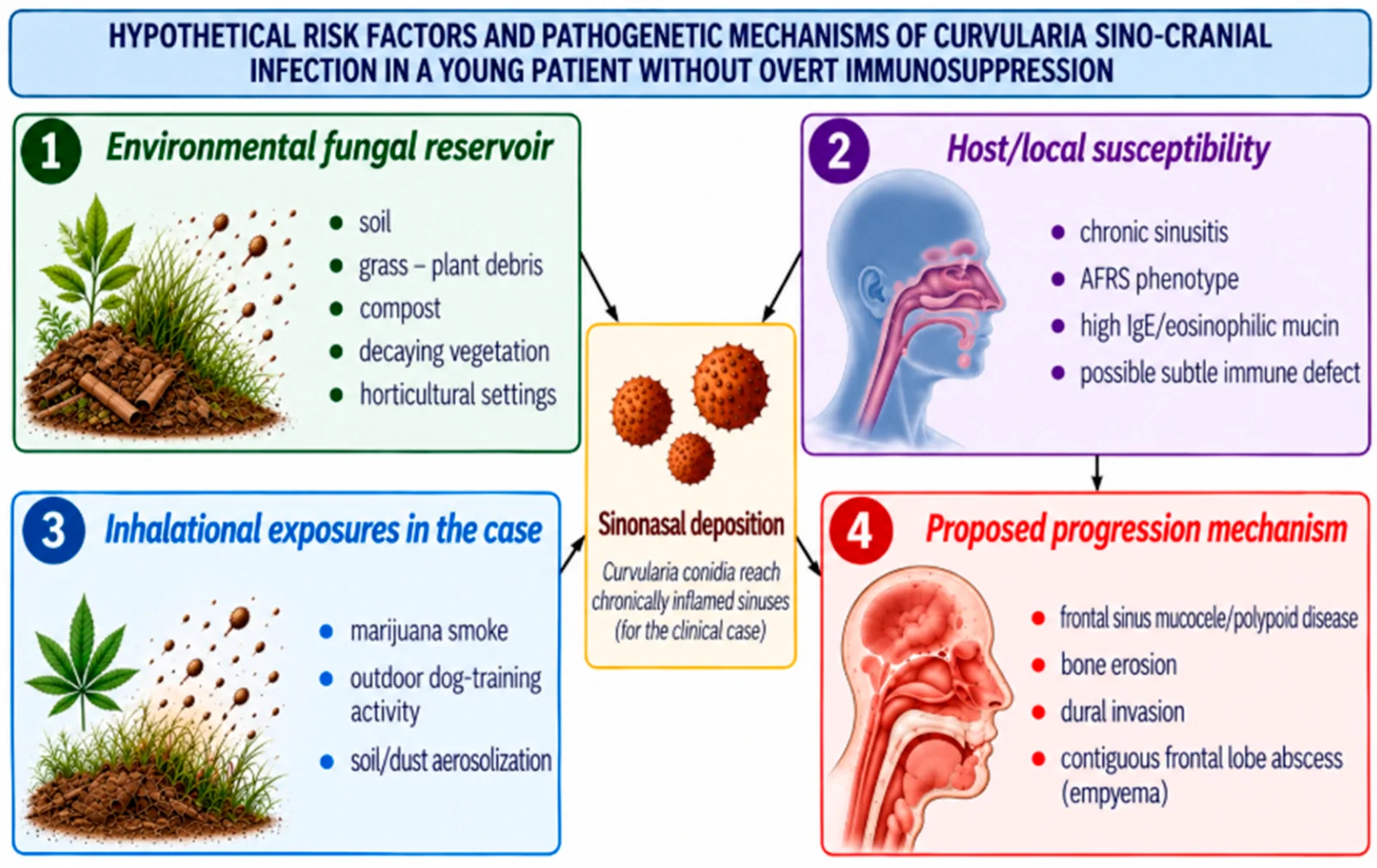
Hypothetical risk factors and proposed mechanisms of *Curvularia* sino-cranial infection in the present clinical case.

**Table 1:** In vitro antifungal susceptibility of the *Curvularia spicifera* isolate.

Antifungal Agent	Initial Laboratory MIC (Microg/mL)	Reference Laboratory MIC (Microg/mL)
Amphotericin B	0.12	<0.12
Anidulafungin	0.25	0.03
Caspofungin	4	4
Micafungin	2	8
Fluconazole	16	64
Itraconazole	0.06	0.03
Voriconazole	0.12	2
Posaconazole	0.01	0.015
Isavuconazole	ND	1
Flucytosine	64	ND

**Table 2:** Published cases of CNS and intracranial *Curvularia* infections.

Patient Age/Sex	Immune Status/Predisposing Factors	CNS and Intracranial/Adjacent Site	Extra CNS Infection	Pathogen	Surgery	Antifungal Therapy	Outcome and Follow-up	Ref.
31/F	Undiagnosed disseminated lymphocytic lymphosarcoma, identified at autopsy	Meningoencephalitis with severe granulomatous vasculitis and a right frontal tumor-like lesion	No	*Drechslera hawaiiensis* (currently classified as *Curvularia hawaiiensis*)	No	None	Died 11 days after surgery	[18]
13/M	Immunocompetent; mild mental disability; eosinophilia/elevated IgE; repeated soil/dirt inhalation	Right frontoparietal cerebral mass; recurrence 3 years later	Pulmonary micetoma	*Curvularia pallescens*	Complete excision; cranial decompression at recurrence	Griseofulvin; amphotericin B stopped for azotemia; IV miconazole for 6 weeks Recurrence treated with IV and intrathecal miconazole	Initial improvement; fatal relapse 3 years later	[19,20]
25/M	Immunocompetent; football player with chronic skin ulcers after football abrasions	Left parietal cerebral abscess	Disseminated to soft-tissue, pulmonary, paravertebral disease	*Curvularia lunata*	Multiple drainage procedures	Miconazole for 5 weeks; later added amphotericin B	Cerebral abscess resolved; systemic relapse	[4]
21/F	Immunocompetent; no clear risk factor	Bilateral frontal cerebral lesions; granulomatous fungal encephalitis	No	*Drechslera spicifera* (currently classified as *Curvularia spicifera*)	Right frontal brain biopsy	Amphotericin B, total dose 2 g, plus flucytosine for 4 months	Survived; persistent hemiparesis and emotional lability	[21]
21/F	Immunocompetent; skull fracture with environmental contamination, persistent CSF leakage and corticosteroid therapy	Post-traumatic meningoencephalitis with fungal vasculitis	No	*Drechslera spicifera* (currently classified as *Curvularia spicifera*)	Wound debridement and craniectomy with dural graft; right frontal lobectomy and evacuation of intracerebral hematoma	No antifungal therapy	Died 28 days after admission	[22]
49/F	Carcinomatous meningitis in previous metastatic breast cancer; Ommaya reservoir for intrathecal chemotherapy	Fungal meningitis and ventriculitis	No	*Bipolaris spicifera* (currently classified as *Curvularia spicifera*)	No	None	Died after 14 days	[23]
26/F	Immunocompetent; chronic sinus infection	Right occipital lesion/meningitis; left temporal and sphenoid wing mass 6 years later	Chronic sinusitis	*B. spicifera* (currently classified as *C. spicifera*)	Craniotomy and biopsy; sinus surgery	Amphotericin B plus ketoconazole for 3 months	Survived with persistent severe visual deficit	[7]
46/F	Immunocompetent; chronic sinusitis	Sphenoidal mass extended intracranially	Chronic sinusitis	*C. lunata*	Trans-septal sphenoidectomy with removal of a fungal ball	Amphotericin B for 4 months, followed by 8 months of oral ketoconazole	No recurrence 2 months after stopping therapy	[24]
26/M	Immunocompetent; nasal polyposis; eosinophilia	Ethmoid/sphenoid allergic fungal sinusitis with orbital extension; Radiological involvement of the cavernous sinus	Noninvasive allergic fungal sinusitis	*Bipolaris spicifera* (currently classified as *Curvularia spicifera*)	Frontoethmoidectomy, orbitotomy; repeat sinus surgery	No antifungal therapy	Asymptomatic at 1 year	[25]
46/F	Immunocompetent; asthma, hay fever, chronic sinusitis; multiple sinus surgeries	Bifrontal cerebritis contiguous with cribriform plate erosion	Invasive sinusitis	*Curvularia clavata*	Bilateral sphenoethmoidectomy and frontal sinusotomy; no brain surgery	Amphotericin B for 7 months, then itraconazole for 1 month	Marked improvement at 24 months	[26]
18/M	Previously healthy; postoperative neurosurgical setting, steroids; recent diving in lake water	Postoperative meningitis	No	*B. spicifera* (currently classified as *C. spicifera*)	No surgery; ventriculostomy for intrathecal therapy	IV and intrathecal amphotericin B plus itraconazole, stopped for liver toxicity: duration 5 months	Survived; residual deficits at 1 year	[27]
21/M	Immunocompetent; asthma; occasional marijuana/cocaine use	Right basal ganglia/anterior horn lesion; later ventricular spread	No	*C. lunata*	Stereotactic needle aspiration and biopsy	Amphotericin B, liposomal amphotericin B; flucytosine added later	Died 1 month after admission	[28]
57/M	Immunocompetent; no clear risk factors	Sphenoid mucocele extending into pituitary fossa/suprasellar region with optic nerve compression	no	*C. lunata*	Transsphenoidal resection	Amphotericin B/liposomal amphotericin B for 4 weeks, then oral voriconazole for 4 weeks	Disease-free at month 8	[29]
35/M	Plasma cell dyscrasia	Large cranial base lesion with brainstem compression and hydrocephalus	No	*Curvularia geniculata*	Transnasal/transclival biopsy and limited resection	Voriconazole; amphotericin B added later	Died 4 months after biopsy	[30]
55/M	Heart transplant 6 weeks earlier; on therapy with mycophenolate, prednisone, cyclosporine	Left basal ganglia abscess	No	*B. spicifera* (currently classified as *C. spicifera*)	Brain biopsy	Liposomal amphotericin B, then voriconazole for 12 months	MRI resolution at 12 months; no relapse after stopping therapy	[31]
50/F	Immunocompetent; chronic sinusitis; previous sinus surgery	Right inferior frontal lobe extending into the ethmoid, right orbit, and cranial base	Sinusitis	*Curvularia* species	Combined CNS and sinus surgery	Voriconazole	Disease-free after 3 years	[32]
37/M	Neurosarcoidosis on chronic steroids; heavy marijuana use	Multiple supratentorial brain abscesses	Pulmonary abscess	*Curvularia* species	Neurosurgical debridement and resection; repeated resection	Empirical IV antifungal therapy, agents not specified	No new lesions at 2-month follow-up	[12]
33/M	Immunocompetent; heavy marijuana use and alcohol abuse	Right medulla/brainstem abscess	Left lung granuloma	*Curvularia* species	First open biopsy and drainage; repeat drainage and subtotal debulking	Voriconazole, flucytosine, liposomal amphotericin B for 10 weeks; prolonged voriconazole (at least 13 months)	Alive and improved at 13 months	[33]
68/M	Previous non-Hodgkin lymphoma treated with chemo-radiotherapy	Multiple supratentorial and subtentorial lesions	No	*Curvularia* species	Brain lesion biopsy	Itraconazole, IV voriconazole, liposomal amphotericin B	Died 10 days after treatment initiation	[34]
43/F	Immunocompetent; intranasal cocaine abuse and perfume inhalant use	Multifocal cerebral infarctions	Kidney abscess, invasive sphenoid sinusitis	*Curvularia buchloes*	Nasal endoscopy and biopsy	IV amphotericin B for 30 days, then switch to oral voriconazole	Neurologic deficits persisted at 30-day follow-up	[35]
28/M	Immunocompetent; chronic rhinosinusitis, nasal polyps, prior sinus surgery, marijuana use	Right anterior cranial fossa/frontal abscess with skull-base extension	Sinusitis	*Curvularia* species	Combined endonasal and bifrontal craniotomy with gross total resection	Amphotericin B 4 weeks, then voriconazole 12 months	Stable at 12-month follow-up	[1]
68/M	Gastric DLBCL treated with chemoradiotherapy and autologous bone marrow transplant 3 years earlier	Multiple bilateral supratentorial necrotizing granulomas		*C. spicifera*	Removal of the most superficial right frontal lesion	Not specified dual IV antifungal therapy	Died 2 months after surgery	[36]
33/F	Immunocompetent; previously treated pulmonary tuberculosis	Chronic invasive sphenoid/ethmoid sinusitis with pituitary, midbrain, facial-vestibulocochlear complex and vascular involvement	Sinusitis	*Curvularia warraberensis*	Endoscopic sinus surgery and bilateral sphenoethmoidectomy	Liposomal amphotericin B plus itraconazole	Died 32 days after admission	[37]

Historical fungal names are reported as originally published. Current nomenclature was updated according to the taxonomic revision of the *Bipolaris–Cochliobolus–Curvularia* complex proposed by Manamgoda et al. and subsequent updates [9,16].

## Data Availability

All relevant data are included in the manuscript. Additional anonymized clinical details may be available from the corresponding author upon reasonable request, subject to institutional policy.

## Abbreviations

The following abbreviations are used in this manuscript:

AFRS	allergic fungal rhinosinusitis
CLSI	Clinical and Laboratory Standards Institute
CNS	central nervous system
CT	computed tomography
ECV	epidemiological cutoff value
MALDI-TOF MS	matrix-assisted laser desorption ionization time-of-flight mass spectrometry
MIC	minimum inhibitory concentration
MRI	magnetic resonance imaging
PK/PD	pharmacokinetic/pharmacodynamic
TDM	therapeutic drug monitoring

## References

[1] Goldman M., Reddy R., Lucke-Wold B., Barpujari A., Cameron M., Porche K., Laurent D., Duarte E., Lobo B.C., Koch M. (2022). Curvularia and the Brain: Case Demonstration of Optimal Management. Arch. Emerg. Med. Crit. Care.

[2] Revankar S.G., Sutton D.A., Rinaldi M.G. (2004). Primary Central Nervous System Phaeohyphomycosis: A Review of 101 Cases. Clin. Infect. Dis..

[3] Ferraro N., Iskandar E., Pitrolo A.M.G., Ramus M., Pagella F., Introini S., Baldanti F., Cavanna C. (2026). Curvularia Spicifera in Non-Invasive Fungal Rhinosinusitis: Case Reports and Diagnostic Insights. Pathogens.

[4] Rohwedder J.J., Simmons J.L., Colfer H., Gatmaitan B. (1979). Disseminated Curvularia Lunata Infection in a Football Player. Arch. Intern. Med..

[5] Velasco J., Revankar S. (2019). CNS Infections Caused by Brown-Black Fungi. J. Fungi.

[6] Kobayashi H., Sano A., Aragane N., Fukuoka M., Tanaka M., Kawaura F., Fukuno Y., Matsuishi E., Hayashi S. (2008). Disseminated Infection by Bipolaris Spicifera in an Immunocompetent Subject. Med. Mycol..

[7] McGinnis M.R., Campbell G., Gourley W.K., Lucia H.L. (1992). Phaeohyphomycosis Caused by Bipolaris Spicifera: An Informative Case. Eur. J. Epidemiol..

[8] Krizsán K., Papp T., Manikandan P., Shobana C.S., Chandrasekaran M., Vágvölgyi C., Kredics L. (2015). Clinical Importance of the Genus Curvularia. Medical Mycology.

[9] Manamgoda D.S., Cai L., McKenzie E.H.C., Crous P.W., Madrid H., Chukeatirote E., Shivas R.G., Tan Y.P., Hyde K.D. (2013). A Phylogenetic and Taxonomic Re-Evaluation of the Bipolaris-Cochliobolus-Curvularia Complex. Fungal Divers..

[10] Revankar S.G., Sutton D.A. (2010). Melanized Fungi in Human Disease. Clin. Microbiol. Rev..

[11] Cavanna C., Seminari E., Pusateri A., Mangione F., Lallitto F., Esposto M.C., Pagella F. (2014). Allergic Fungal Rhinosinusitis Due to Curvularia Lunata. New Microbiol..

[12] Gongidi P., Sarkar D., Behling E., Brody J. (2013). Cerebral Phaeohyphomycosis in a Patient with Neurosarcoidosis on Chronic Steroid Therapy Secondary to Recreational Marijuana Usage. Case Rep. Radiol..

[13] Chowdhary A., Meis J.F., Guarro J., de Hoog G.S., Kathuria S., Arendrup M.C., Arikan-Akdagli S., Akova M., Boekhout T., Caira M. (2014). ESCMID and ECMM Joint Clinical Guidelines for the Diagnosis and Management of Systemic Phaeohyphomycosis: Diseases Caused by Black Fungi. Clin. Microbiol. Infect..

[14] CLSI M61-Performance Standards for Antifungal Susceptibility Testing of Filamentous Fungi|GlobalSpec. https://standards.globalspec.com/std/14297791/clsi-m61.

[15] Patel R., Mendrick C., Knapp C.C., Grist R., McNicholas P.M. (2007). Clinical Evaluation of the Sensititre YeastOne Plate for Testing Susceptibility of Filamentous Fungi to Posaconazole. J. Clin. Microbiol..

[16] Kidd S.E., Westblade L.F. (2024). Bipolaris or Curvularia? Resolving the Spicy Issue of How Clinical Isolates Should Be Reported. PLoS Pathog..

[17] EUCAST: Clinical Breakpoint Tables. https://www.eucast.org/bacteria/clinical-breakpoints-and-interpretation/clinical-breakpoint-tables/.

[18] Fuste F.J., Ajello L., Threlkeld R., Henry J.E. (1973). Drechslera Hawaiiensis: Causative Agent of a Fatal Fungal Meningo-Encephalitis. Med. Mycol..

[19] Lampert R.P., Hutto J.H., Donnelly W.H., Shulman S.T. (1977). Pulmonary and Cerebral Mycetoma Caused by Curvularia Pallescens. J. Pediatr..

[20] Friedman A.D., Campos J.M., Rorke L.B., Bruce D.A., Arbeter A.M. (1981). Fatal Recurrent Curvularia Brain Abscess. J. Pediatr..

[21] Yoshimori R.N., Moore R.A., Itabashi H.H., Fujikawa D.G. (1982). Phaeohyphomycosis of Brain: Granulomatous Encephalitis Caused by Drechslera Spicifera. Am. J. Clin. Pathol..

[22] Biggs P.J., Allen R.L., Powers J.M., Holley H.P. (1986). Phaeohyphomycosis Complicating Compound Skull Fracture. Surg. Neurol..

[23] Adam R.D., Paquin M.L., Petersen E.A., Saubolle M.A., Rinaldi M.G., Corcoran J.G., Galgiani J.N., Sobonya R.E. (1986). Phaeohyphomycosis Caused by the Fungal Genera Bipolaris and Exserohilum. A Report of 9 Cases and Review of the Literature. Medicine.

[24] Ismail Y., Johnson R.H., Wells M.V., Pusavat J., Douglas K., Arsura E.L. (1993). Invasive Sinusitis with Intracranial Extension Caused by Curvularia Lunata. Arch. Intern. Med..

[25] Klapper S.R., Lee A.G., Patrinely J.R., Stewart M., Alford E.L. (1997). Orbital Involvement in Allergic Fungal Sinusitis. Ophthalmology.

[26] Ebright J.R., Chandrasekar P.H., Marks S., Fairfax M.R., Aneziokoro A., McGinnis M.R. (1999). Invasive Sinusitis and Cerebritis Due to Curvularia Clavata in an Immunocompetent Adult. Clin. Infect. Dis..

[27] Latham R.H. (2000). Bipolaris Spicifera Meningitis Complicating a Neurosurgerical Procedure. Scand. J. Infect. Dis..

[28] Carter E., Boudreaux C. (2004). Fatal Cerebral Phaeohyphomycosis Due to Curvularia Lunata in an Immunocompetent Patient. J. Clin. Microbiol..

[29] Smith T., Goldschlager T., Mott N., Robertson T., Campbell S. (2007). Optic Atrophy Due to Curvularia Lunata Mucocoele. Pituitary.

[30] Singh H., Irwin S., Falowski S., Rosen M., Kenyon L., Jungkind D., Evans J. (2008). Curvularia Fungi Presenting as a Large Cranial Base Meningioma: Case Report. Neurosurgery.

[31] Rosow L., Jiang J.X., Deuel T., Lechpammer M., Zamani A.A., Milner D.A., Folkerth R., Marty F.M., Kesari S. (2011). Cerebral Phaeohyphomycosis Caused by Bipolaris Spicifera after Heart Transplantation. Transpl. Infect. Dis..

[32] Gadgil N., Kupferman M., Smitherman S., Fuller G.N., Rao G. (2013). Curvularia Brain Abscess. J. Clin. Neurosci..

[33] Skovrlj B., Haghighi M., Smethurst M.E., Caridi J., Bederson J.B. (2014). Curvularia Abscess of the Brainstem. World Neurosurg..

[34] Bova C., Vigna E., Gentile M., Fiaschi E. (2022). Cerebral Pheohyphomycosis Due to Curvularia Species. IDCases.

[35] Pham J., Kulla B., Johnson M. (2022). Invasive Fungal Infection Caused by Curvularia Species in a Patient with Intranasal Drug Use: A Case Report. Med. Mycol. Case Rep..

[36] Bono B.C., Olei S., Rossini Z., Fernandes B., Valeri M., Pessina F. (2022). Primary Brain Necrotizing Granulomas Caused by Curvularia Spicifera. Infect. Dis. Now..

[37] Samaddar A., Shrimali T., Tiwari S., Sharma A. (2023). First Report of Human Infection Caused by Curvularia Warraberensis, Manifesting as Invasive Sinusitis with Intracranial Involvement. J. Mycol. Med..

[38] Posteraro B., Scarano E., La Sorda M., Torelli R., De Corso E., Mulé A., Paludetti G., Fadda G., Sanguinetti M. (2010). Eosinophilic Fungal Rhinosinusitis Due to the Unusual Pathogen Curvularia Inaequalis. Mycoses.

[39] Riegler A.N., Grayson J.W., Greenway J.W., Woodworth B.A., McCarty T.P., Pappas P.G., Leal S.M. (2025). Histopathologic Insights Into Curvularia-Induced Chronic Granulomatous Fungal Sinusitis: A Guide for Surgical Pathologists. Mod. Pathol..

[40] Marinucci V., Chahine E.B., Bush L.M. (2022). Invasive Fungal Rhinosinusitis Caused by Curvularia in a Patient With Type 1 Diabetes Mellitus: A Case Report. J. Pharm. Pr..

[41] Gwinn K.D., Leung M.C.K., Stephens A.B., Punja Z.K. (2023). Fungal and Mycotoxin Contaminants in Cannabis and Hemp Flowers: Implications for Consumer Health and Directions for Further Research. Front. Microbiol..

[42] Rothenburg L.S., Snider T.A., Wilson A., Confer A.W., Ramachandran A., Mani R., Rizzi T., Nafe L. (2017). Disseminated Phaeohyphomycosis in a Dog. Med. Mycol. Case Rep..

[43] Jaffey J.A., Cañete-Gibas C.F., Wiederhold N.P., Sanders C.J., Struthers J.D., Black A., Wu B., Thomas K.S., Bennett P., Watt J. (2025). Novel Curvularia Species Causing Disseminated Phaeohyphomycosis in a Dog. Top. Companion Anim. Med..

[44] Herráez P., Rees C., Dunstan R. (2001). Invasive Phaeohyphomycosis Caused by Curvularia Species in a Dog. Vet. Pathol..

[45] da Cunha K.C., Sutton D.A., Fothergill A.W., Gené J., Cano J., Madrid H., de Hoog S., Crous P.W., Guarro J. (2013). In vitro antifungal susceptibility and molecular identity of 99 clinical isolates of the opportunistic fungal genus Curvularia. Diagn. Microbiol. Infect. Dis..

[46] Espinel-Ingroff A. (2001). In Vitro Fungicidal Activities of Voriconazole, Itraconazole, and Amphotericin B against Opportunistic Moniliaceous and Dematiaceous Fungi. J. Clin. Microbiol..

[47] Zheng H., Song N., Mei H., Dong J., Li D., Li X., Liu W. (2020). In Vitro Activities of Ravuconazole and Isavuconazole against Dematiaceous Fungi. Antimicrob. Agents Chemother..

[48] Cornely O.A., Mullane K.M., Ostrosky-Zeichner L., Maher R.M., Croos-Dabrera R., Lu Q., Lademacher C., Perfect J.R., Oren I., Schmitt-Hoffmann A.-H. (2018). Isavuconazole for Treatment of Rare Invasive Fungal Diseases. Mycoses.

[49] Bengyella L., Yekwa L.E., Waikhom S.D., Nawaz K., Iftikhar S., Motloi T.S., Tambo E., Roy P. (2017). Upsurge in *Curvularia* Infections and Global Emerging Antifungal Drug Resistance. Asian J. Sci. Res..

